# Progressive Proteome Changes in the Myocardium of a Pig Model for Duchenne Muscular Dystrophy

**DOI:** 10.1016/j.isci.2020.101516

**Published:** 2020-09-01

**Authors:** Hathaichanok Tamiyakul, Elisabeth Kemter, Miwako Kösters, Stefanie Ebner, Andreas Blutke, Nikolai Klymiuk, Florian Flenkenthaler, Eckhard Wolf, Georg J. Arnold, Thomas Fröhlich

**Affiliations:** 1 Laboratory for Functional Genome Analysis, LAFUGA, Gene Center, LMU Munich, 81377 Munich, Germany; 2 Institute of Molecular Animal Breeding and Biotechnology, Gene Center and Department of Veterinary Sciences, LMU Munich, 81377 Munich, Germany; 3 Center for Innovative Medical Models (CiMM), LMU Munich, 85764 Oberschleißheim, Germany; 4 Institute of Experimental Genetics, Helmholtz Zentrum München, German Research Center for Environmental Health, 85764 Neuherberg, Germany

**Keywords:** Pathophysiology, Proteomics

## Abstract

Duchenne muscular dystrophy (DMD), caused by mutations in the dystrophin gene, is characterized by progressive muscle weakness. Even though DMD manifests first in skeletal muscle, heart failure is a major cause of death in late-stage DMD. To get insights into DMD-associated cardiomyopathy, we performed a proteome analysis of myocardium from a genetically engineered porcine DMD model resembling clinical and pathological hallmarks of human DMD. To capture DMD progression, samples from 2-day- and 3-month-old animals were analyzed. Dystrophin was absent in all DMD samples, and components of the dystrophin-associated protein complex were decreased, suggesting destabilization of the cardiomyocyte plasma membrane and impaired cellular signaling. Furthermore, abundance alterations of proteins known to be associated with human cardiomyopathy were observed. Compared with data from skeletal muscle, we found clear evidence that DMD progression in myocardium is not only slower than in skeletal muscle but also involves different biological and biochemical pathways.

## Highlights

• Proteomics of myocardium from a genetically engineered DMD pig model was performed
• The results indicate an arising inflammatory pathology in the myocardium of DMD pigs
• Alterations of proteins known to be related to cardiomyopathy were found in DMD pigs
• Proteome changes in DMD pigs markedly differ between myocardium and skeletal muscle

## Graphical Abstract

**Figure:**
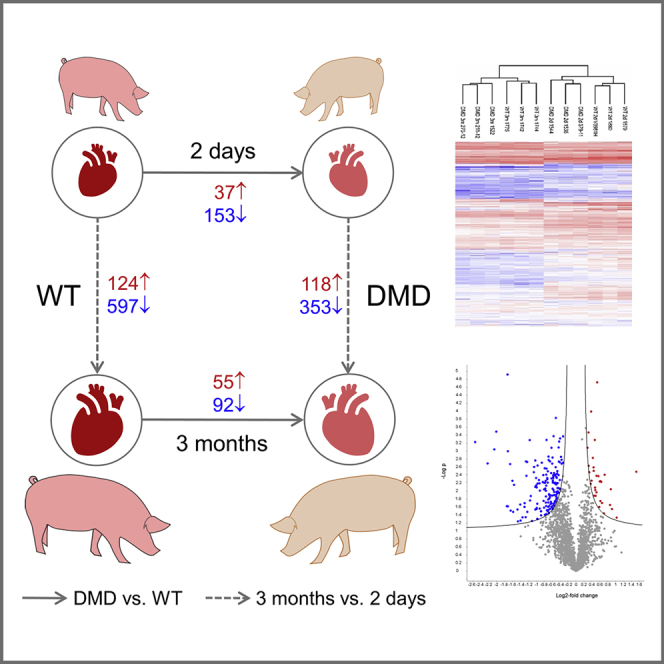

## Introduction

Duchenne muscular dystrophy (DMD) is the most common genetic muscle disease with pediatric onset. Birth prevalence was reported to range from 15.9 to 19.5 per 100,000 live births (Ryder et al., 2017). DMD is caused by loss-of-function mutations of the dystrophin (*DMD*) gene, located on the X chromosome. Dystrophin is part of the dystrophin-associated protein complex (DAPC), which connects the muscle fiber cytoskeleton through the cell membrane to the extracellular matrix. In DMD, the absence of dystrophin results in progressive muscle degeneration. Male patients are mostly diagnosed around 5 years of age and lose their ability to walk before their teens (Ryder et al., 2017). Without suitable therapy, the mean age at death is around 19 years. For a comprehensive review please refer to Birnkrant et al. (2018) and Bushby et al. (2010). Even though DMD manifests first in skeletal muscle, DMD is a multi-system disease also affecting the heart, especially in the second decade of life. DMD-related cardiomyopathy has a prevalence of nearly one-third in patients aged 14 years and is observed in around one-half of 18-year-old patients (Shih et al., 2020). The typical pathology includes arrhythmias, systolic dysfunction, and dilated cardiomyopathy associated with fibrosis of the inferior and inferolateral walls of the left ventricle (for a detailed review please refer to Meyers and Townsend, 2019). Especially with improvements in respiratory management, heart failure is increasingly becoming the main cause of morbidity and mortality of patients with DMD (Hor et al., 2018). Although dystrophic cardiomyopathy is known to be related to cardiomyocyte membrane instability, dysfunction of ion channels, impaired calcium handling, inflammatory response, and fibrosis, its molecular pathogenesis is not well understood. To mimic the disease and to allow studies at the molecular level, a variety of animal models have become available among which the *mdx* mouse (Sicinski et al., 1989) is the most commonly used (reviewed in Nakamura and Takeda, 2011). At the proteome level, several studies addressed the heart of *mdx* mice to characterize disturbances associated with dystrophin deficiency (Holland and Ohlendieck, 2014). A combined metabolomic and proteomic investigation of heart tissue from *mdx* mice revealed increased taurine levels and increased abundances of proteins related to oxidative phosphorylation and mitochondrial metabolism (Gulston et al., 2008). A further proteomics study showed that in the heart tissue of *mdx* mice, the lack of dystrophin led to altered levels of proteins related to energy metabolism and the contractile apparatus (Lewis et al., 2010). Another study analyzed heart tissue from *mdx-4cv* mice and found proteins involved in muscle contraction, energy metabolism, signaling, and stress response to be altered in abundance (Murphy et al., 2016).

**Figure 1:**
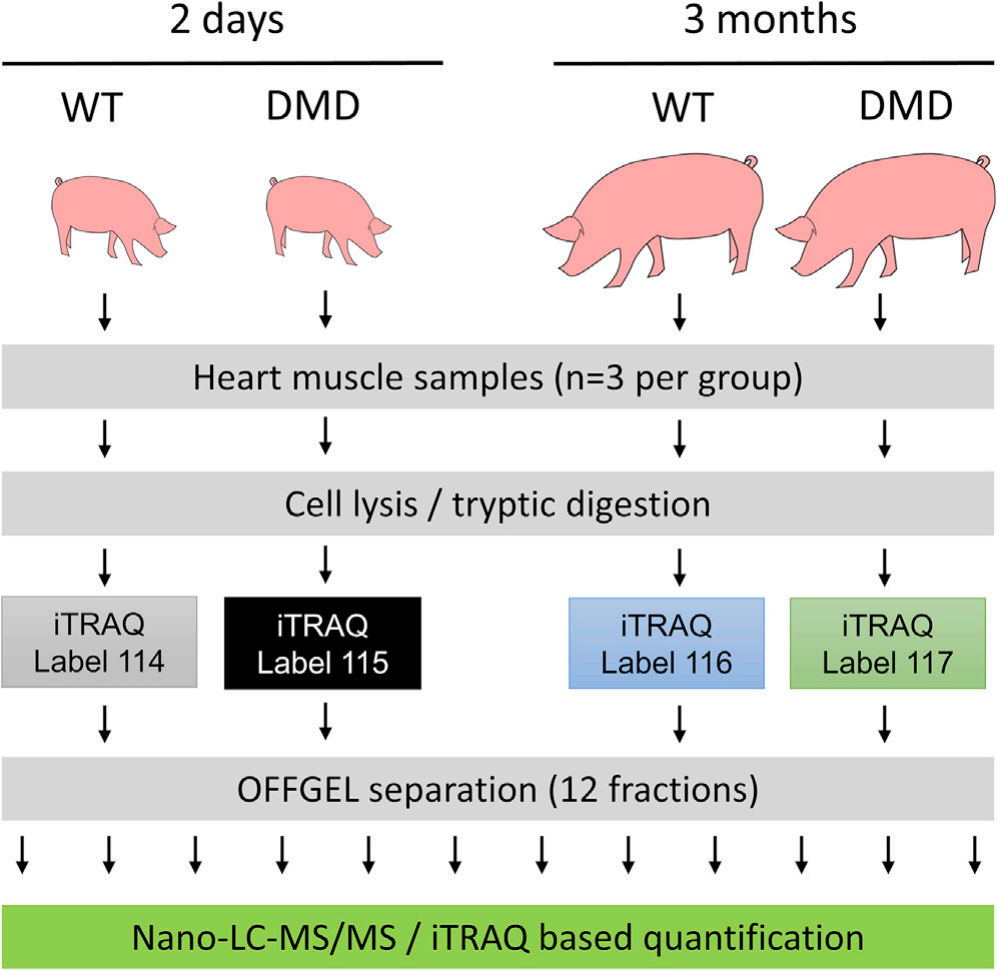
Overview of the Experimental Strategy An iTRAQ-based quantification strategy and OFFGEL pre-fractionation was used.

Although *mdx* mice have been proved to be valuable models to study the biochemistry of DMD, this model has only a slightly shorter life span compared with wild-type (WT) mice and shows, with the exception of the diaphragm, no severe muscle pathology (Partridge, 2013; Wells, 2018). Clinically more relevant muscle pathologies are observed in larger animal models for DMD, like dog and pig models (reviewed in Yu et al., 2015). For this study, we chose a pig model of DMD, which lacks *DMD* exon 52 and thus resembles a frequent mutation in human DMD (Klymiuk et al., 2013). The model shows clinical signs of DMD, like a severe myopathy, elevated serum creatine kinase levels, a progressively impaired mobility, and a shortened life expectancy of about 3 months. In a prior study, we used this pig model to investigate proteome alterations in skeletal muscle of young (2-day-old) and older (3-month-old) DMD pigs (Frohlich et al., 2016), demonstrating that this model reflects the progressively developing biochemical and histological hallmarks of the human disease. More recently, the DMD pig model was instrumental for validating multispectral optoacoustic tomography as an imaging biomarker for progressive muscle fibrosis in DMD (Regensburger et al., 2019) and for testing a CRISPR/Cas9-based therapeutic approach aiming at the restoration of an intact *DMD* reading frame (Moretti et al., 2020). The present study uses a well-defined set of heart tissue samples from this pig model to investigate age-related proteome alterations in the myocardium caused by the lack of dystrophin. The experimental design is shown in Figure 1. Moreover, proteome alterations in the myocardium are compared with those in skeletal muscle tissue from the same animals.

## Results

### Pathological and Histomorphological Findings

**Figure 2:**
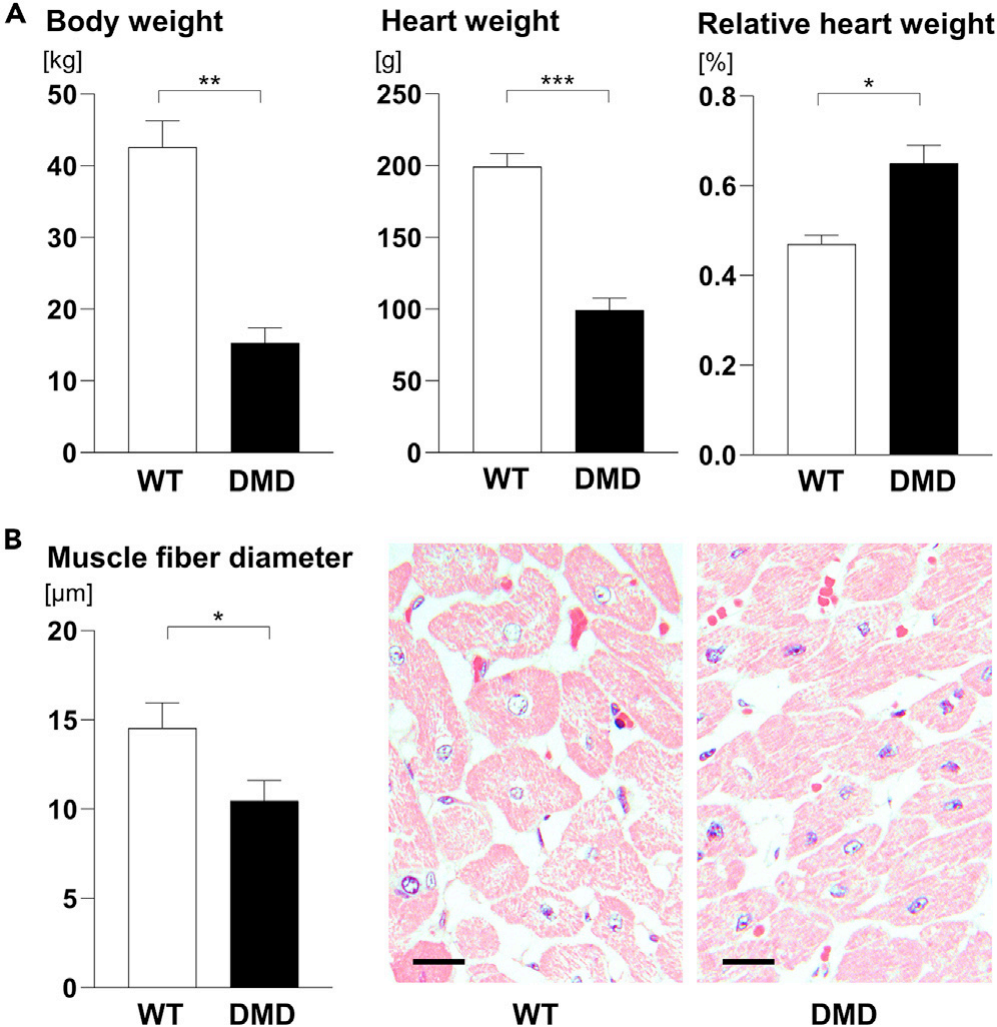
Heart Weights and Muscle Fiber Diameters of 3-Month-Old DMD Animals Compered with WT (A) Body weights and absolute and relative heart weights of WT and DMD pigs at 3 months of age. (B) Mean minimal muscle fiber diameters and representative histology of the papillary muscle of the left ventricle in 3-month-old WT and DMD pigs. Methacrylate and methyl methacrylate (GMA/MMA)- sections, hematoxylin and eosin staining; scale bars, 10 μm. Data are means ± standard deviations. Significant differences (Student's t test) are indicated by asterisks. ∗p < 0.05, ∗∗p < 0.01, ∗∗∗p < 0.001.

The body weights of 2-day-old DMD (1.32 ± 0.25 kg) and WT piglets (1.22 ± 0.19 kg) were not significantly different (p = 0.61), whereas 3-month-old DMD pigs displayed significantly lower body weights (as earlier reported by Klymiuk et al., 2013) and absolute heart weights than WT controls. In contrast, the relative heart weights were significantly higher in DMD pigs (Figure 2A). Furthermore, the mean muscle fiber diameters of the left ventricular papillary muscle were significantly reduced in 3-month-old DMD pigs, when compared with WT controls (Figure 2B).

### Overview of Identified Proteins and Proteome Changes

**Figure 3:**
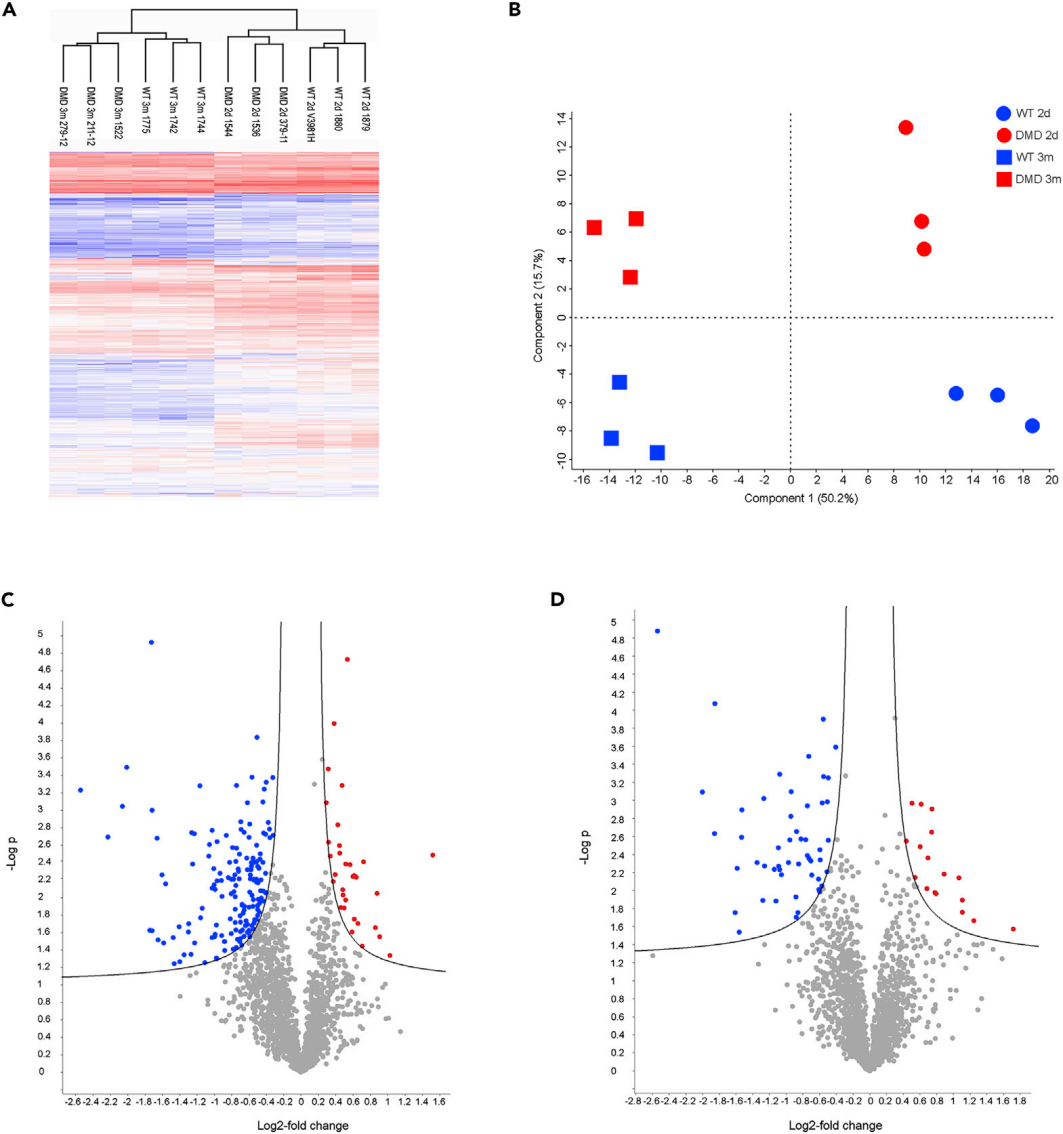
Aberration of the Heart Proteome of DMD Pigs (A–D) Both (A) heatmap analysis and (B) principal-component analysis (PCA) of iTRAQ intensity values clearly separate age and genotypes. Volcano plot analysis of DMD versus WT proteomes of (C) 2-day-old and (D) 3-month-old animals indicates significant alterations between proteomes of the heart muscle of DMD and WT animals. Proteins differentially more abundant (p < 0.05) in DMD heart are highlighted in red, proteins and less abundant in DMD are highlighted in blue.

**Figure 4:**
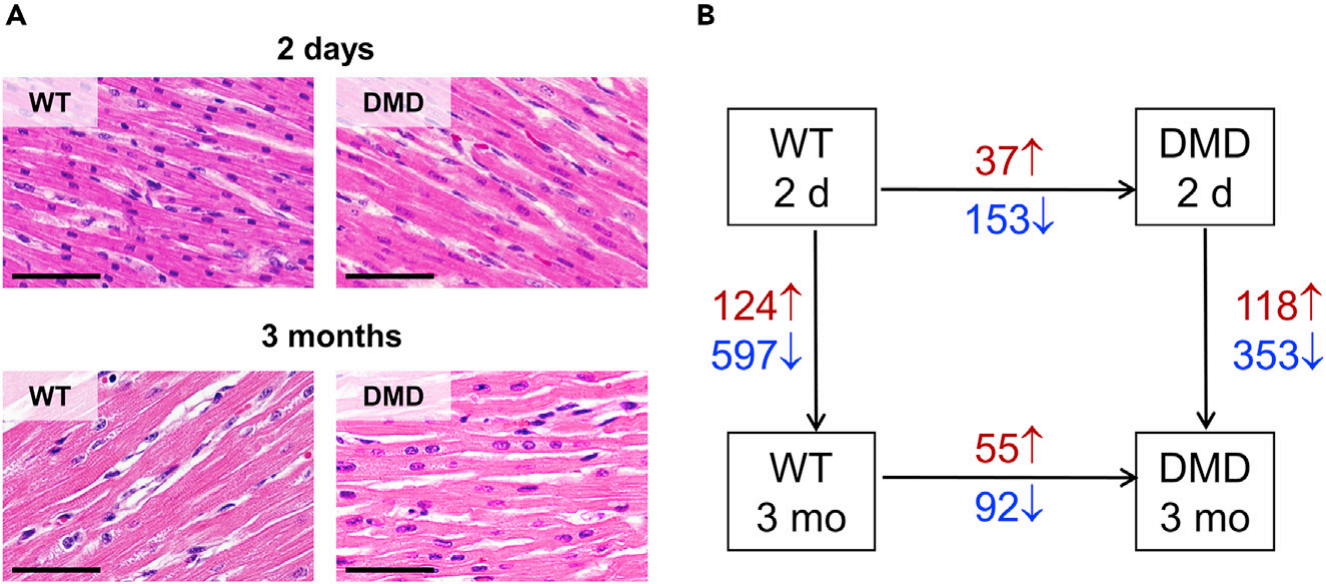
Histology and Proteome Changes in the Myocardium of DMD Pigs (A and B) (A) Representative myocardial histology (left ventricle) of WT and DMD pigs at 2 days and 3 months of age. (GMA/MMA sections; hematoxylin and eosin staining; scale bars, 50 μm). (B) Numbers of significantly increased (red) and decreased (blue) proteins (p value < 0.05; log2-fold change > |0.6|) for the comparisons DMD versus WT animals within age (horizontal arrows) and 3-month versus 2-day-old animals within genotype (vertical arrows).

We performed an iTRAQ 4plex-based liquid chromatography-tandem mass spectrometry (LC-MS/MS)-based approach to analyze heart tissue samples from 2-day-old and 3-month-old DMD and WT pigs (Figure 1). As several proteins are highly abundant in the heart muscle (e.g., titin, myosin 7 and several actins), which weakens the analytical depth of single LC-MS/MS runs, we performed an OFFGEL-based prefractionation at the peptide level. In total, we were able to quantify 19,509 different peptides belonging to 2,450 proteins with at least two individual peptides. A list of all identified proteins can be found in Table S1. A PANTHER functional classification analysis revealed a broad functional spectrum of the identified proteins. A pie chart summarizing the result is shown as Figure S1. Hierarchical clustering of the normalized iTRAQ signal intensity values (Figure 3A) as well as a principal-component analysis (Figure 3B) clearly separated all groups according to age and genotype. Furthermore, a volcano plot analysis revealed significant differences between the heart muscle proteome of WT and DMD pigs (Figures 3C and 3D). The histology and degree of proteome changes in the myocardium of DMD pigs is summarized in Figure 4. Representative histological images of the investigated heart samples are shown in Figure 4A. The numbers of differentially abundant proteins (adjusted p value < 0.05; log2-fold change > |0.6|) are shown in Figure 4B. Beside proteome alterations directly or indirectly caused by the lack of dystrophin, strong age-dependent changes in the heart muscle proteomes were observed.

### Age-Dependent Proteome Differences in WT and DMD Pigs

Growth of WT and DMD hearts was reflected by a large number of proteins altered in abundance when comparing 3-month-old and 2-day-old animals. In WT pigs, 597 proteins were found to be less abundant and 124 more abundant in heart tissue from 3-month-old versus 2-day-old animals. The corresponding numbers of differentially abundant proteins in DMD pigs were 353 and 118. Proteomaps analyses revealed that among the proteins being more abundant in the myocardium of young animals, many are related to transcription and translation (see Figure S2A for WT and Figure S3A for DMD). This may reflect a higher demand of newly synthesized transcripts and proteins during rapid growth of the heart in the postnatal period. The majority of proteins more abundant in the heart muscle of older animals are related to energy metabolism and amino acid as well as lipid/steroid metabolism (see Figure S2B for WT and Figure S3B for DMD). The lists of proteins with age-dependent abundance changes in WT and DMD myocardium are provided in Tables S2 and S3.

### Differentially Abundant Proteins in 2-Day-Old DMD versus WT Pig Myocardium

**Figure 5:**
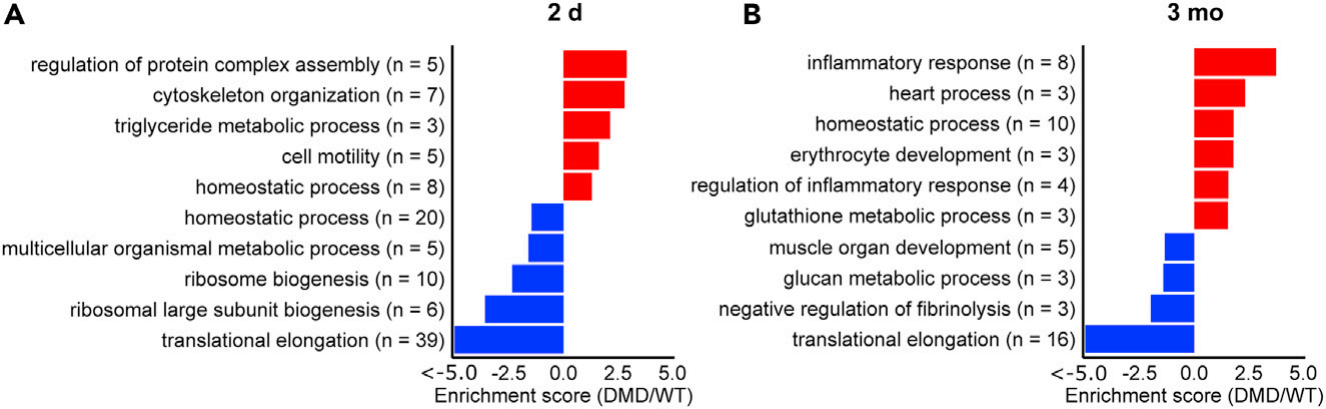
Functional Annotation Clustering (A and B) DAVID annotation clusters with an enrichment score >1.3 for proteins more abundant (positive enrichment scores, red) or less abundant (negative enrichment scores, blue) in (A) 2-day-old and (B) 3-month-old DMD pigs compared with age-matched WT animals.

Analysis of data from 2-day-old DMD pigs and age-matched WT animals led to 190 proteins being altered in abundance, of which 153 were less abundant and 37 more abundant in DMD myocardium (Table S4). DAVID analysis of the proteins with decreased abundance revealed five annotation clusters with an enrichment score >1.3 (Figure 5A and Table S6). The by far most prominent one (enrichment score: 44.0) was related to translation and contained 35 ribosomal proteins. In addition, several muscle-related proteins, like syntrophin alpha 1 (SNTA1), myosin 6 (LOC100736765), myosin regulatory light chain 2 (MYL7), and the two creatine kinases CKM and CKB, were found to be significantly reduced. DAVID analysis of proteins being more abundant in DMD myocardium revealed five significant annotation clusters (enrichment score >1.3), of which the most prominent were related to regulation of protein complex assembly (five proteins, enrichment score: 2.9) and regulation of cytoskeleton organization (seven proteins, enrichment score: 2.8) (Figures 5A and Table S7). Among them, spectrin alpha 2 (SPTA1), spectrin beta chain (SPTB), and ankyrin-1 (ANK1) were found. In addition, several proteins directly linked to the sarcomeres, like keratin 8 (KRT8), keratin 19 (KRT19), caveolin 3 (CAV3), and myotilin (MYOT), were found to be more abundant in the heart muscle of 2-day-old DMD versus WT pigs.

### Differentially Abundant Proteins in 3-Month-Old DMD versus WT Pig Myocardium

The quantitative comparison of myocardial proteomes from 3-month-old DMD pigs and age-matched WT animals led to the detection of 147 differentially abundant proteins, of which 92 were less abundant and 55 more abundant in the DMD samples (Table S5). DAVID analysis of the proteins with decreased abundance revealed four annotation clusters with an enrichment score >1.3 (Figures 5B and Table S8), of which, like in the 2-day-old animals, the most prominent (enrichment score: 13.8) is related to translation and ribosomes. However, unlike in 2-day-old animals, beside dystrophin and SNTA1, the levels of several sarcoglycans (SGCA, SGCB, and SGCD) and dystrobrevin alpha (DTNA) were found to be decreased in 3-month-old DMD animals. The DAVID analysis of proteins more abundant in 3-month-old DMD myocardium revealed six annotation clusters with enrichment scores >1.3, of which several were not enriched in the comparison of the 2-day-old animals (see Figure 5B). Interestingly, the most prominent cluster (8 proteins, enrichment score 3.8) contained proteins related to inflammatory response, i.e., alpha-1-acid glycoprotein 1 (ORM1), complement component C7 (C7), alpha-2-macroglobulin (A2M), protein S100-A8 (S100A8), Toll-interacting protein (TOLLIP), inter-alpha-trypsin inhibitor heavy chain H4 (ITIH4), the platelet-activating factor acetylhydrolase (PLA2G7), and alpha-2-HS-glycoprotein (AHSG). Furthermore, similar to the 2-day-old animals, several proteins related to cardiac muscle contraction, i.e., KRT8, MYL7, and myosin light chain 4 (MYL4) were found to be increased in the myocardium of 3-month-old DMD animals.

### Immunohistochemical Findings in the Heart

**Figure 6:**
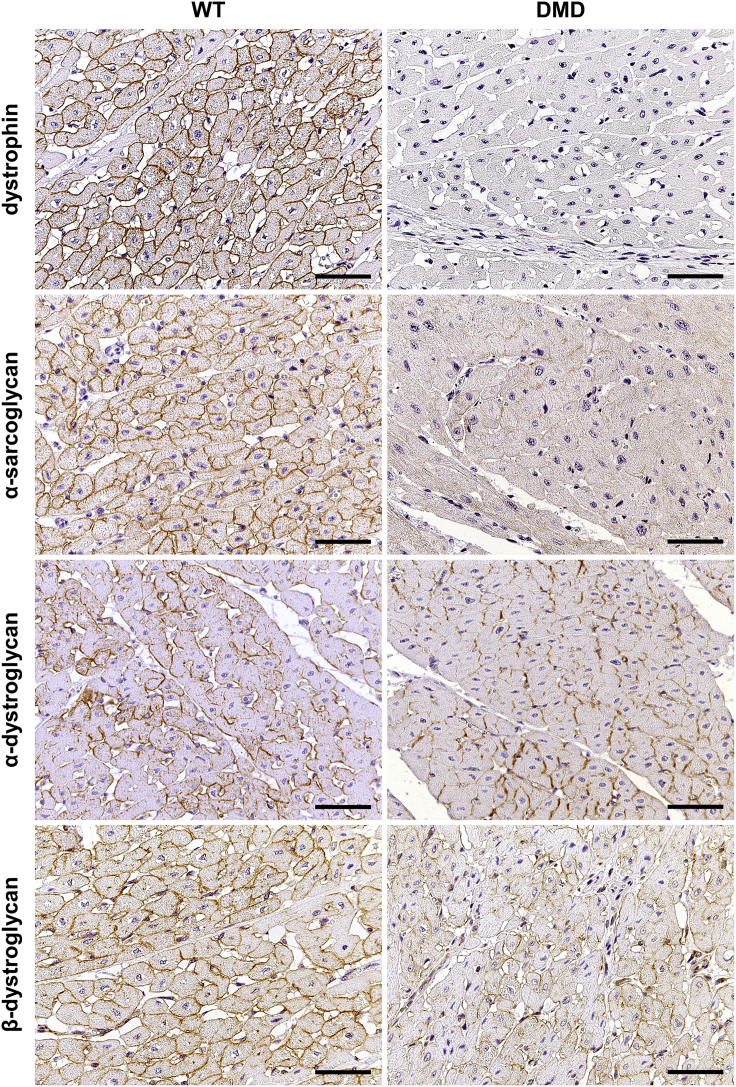
Immunohistochemical Study of Members of the Dystrophin-Associated Protein Complex (DAPC) in the Myocardium of 3-Month-Old DMD and WT Pig Paraffin sections, chromogen: 3,3′-diaminobenzidine (DAB, brown color), nuclear counterstain: hemalum, scale bars, 50 μm.

**Figure 7:**
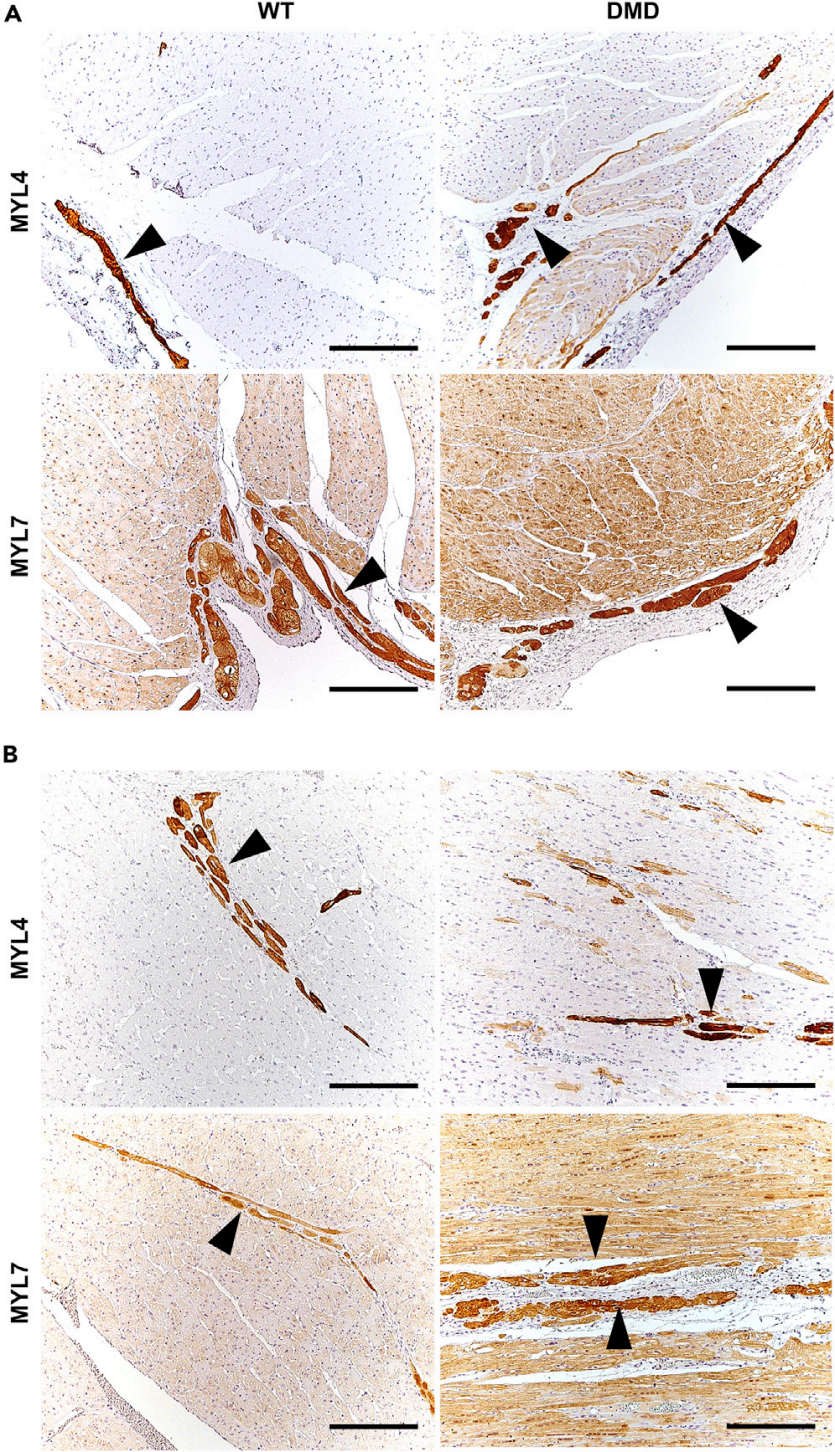
Immunohistochemical Demonstration of MYL4 and MYL7 Expression Pattern in the Myocardium of 3-Month-Old DMD and WT Pigs (A and B) Shown are endocardial area (A) and center (B) of the ventricle myocardium. Arrowheads indicate Purkinje cells. Paraffin sections, chromogen: 3,3′-diaminobenzidine (DAB, brown color), nuclear counterstain: hemalum, scale bars, 200 μm.

Most cardiomyocytes of WT pigs exhibited strong membranous dystrophin staining, which was constantly absent in heart muscle cells of DMD pigs (Figure 6). To study further members of the DAPC, α-sarcoglycan, α-dystroglycan, and β-dystroglycan were selected. In 3-month-old WT pigs, α-sarcoglycan was strongly present at the cell membranes of heart muscle cells. In contrast, only a faint α-sarcoglycan membrane staining was detectable in cardiomyocytes of age-matched DMD animals. Both α-dystroglycan and β-dystroglycan were present in various staining intensities at cardiomyocyte membranes of WT pigs and appeared to be more heterogeneous and partially weaker in heart muscle cells of age-matched DMD animals. In addition, we performed immunohistochemistry studies of MYL4 and MYL7 (Figure 7), for which the proteome analysis revealed an increased abundance in 3-month-old DMD versus WT heart ventricle samples. In ventricle samples of WT animals, MYL4 expression was restricted to Purkinje fibers. In contrast, beside strong MYL4 expression in Purkinje cells, some ventricle cardiomyocytes of DMD pigs exhibited MYL4 expression, predominantly near the endocardial site but occasionally also scattered within ventricle muscle. MYL7 expression was observed in Purkinje fibers and most cardiomyocytes in heart ventricle of 3-month-old WT pigs. Here ventricle of DMD pigs exhibited similar MYL7 staining pattern as observed in age-matched WT pig heart, but staining of MYL7 was more intense in cardiomyocytes of the DMD heart. Differences of immunohistochemical (IHC) signal intensities were quantified by digital image analyses (Figures S4 and S5). In line with the mass spectrometry data, the optical densities of DMD-myocardium sections stained by IHC for proteins of the DAPC (i.e., α-sarcoglycan, α-dystroglycan, and β-dystroglycan) were significantly reduced, when compared with myocardium sections of WT pigs. Also in line with the MS data, the MYL4- and MYL7-IHC staining intensities in myocardium sections of DMD pigs were significantly higher than in WT pigs. To study the fascia adherens/intercalated disks of cardiomyocytes, we selected γ-catenin and observed no obvious difference between 3-month-old DMD and WT pigs (Figure S6).

## Discussion

### General Aspects

Although the primary symptom of DMD is a progressive weakness of skeletal muscles ongoing with massive changes of tissue function and integrity, patients with DMD frequently develop cardiomyopathies in the second and third decade of life (for comprehensive reviews please refer to D'Amario et al., 2017; Hor et al., 2018; Kamdar and Garry, 2016; Meyers and Townsend, 2019; Townsend et al., 2007). Even though end-stage heart failure has become a major cause of death in DMD, the molecular pathology is poorly understood. However, a deeper understanding of the underlying biochemical mechanisms is crucial for the development of new treatment strategies slowing down the progression of DMD-associated cardiomyopathy and supporting heart function in these patients. In this context, the comparative proteome analysis of dystrophin-deficient versus healthy heart tissue is a very promising approach. As sampling of myocardium from human patients is risky and thus not routinely feasible, the usage of suitable animal models is unavoidable. In this study, we used samples of myocardium from a DMD pig model, which lacks *DMD* exon 52 (Klymiuk et al., 2013) and shows a severe clinical phenotype. In skeletal muscle, it was demonstrated that this large animal model reflects the hallmarks of human DMD at the proteome level (Frohlich et al., 2016). To investigate the progression of proteome derangements caused by the lack of dystrophin in the heart, we used tissues from 2-day-old animals, representing early-stage DMD, as well as tissues from 3-month-old animals, reflecting a more advanced stage. As the goal of this study was to address molecular pathways involved in the development of DMD-related cardiomyopathy, animals aged three months were well suited because no advanced histopathological lesions in the heart were observable at this stage (Klymiuk et al., 2013), but functional alterations were already observed by electrophysiological mapping (Moretti et al., 2020).

As proteomics of heart tissue is challenging due to several highly abundant proteins frequently preventing the quantification of lower abundant proteins, we used OFFGEL pre-fractionation. To detect smaller differences between DMD and WT and to prevent the variation during pre-fractionation, we used an iTRAQ approach allowing to pool samples of all four experimental groups before OFFGEL pre-fractionation. Although the OFFGEL technique, which is based on isoelectric focusing, frequently leads to the loss of high-molecular-weight proteins as well as very basic and very acidic proteins, this effect was at least partially compensated because we performed the separation after trypsin digestion, mostly leading to peptides with suitable molecular weight and isoelectric point. The analytical depth of the generated dataset (2,450 quantified proteins) was considerably high. As expected, typical proteins of the sarcomere (e.g., titin, nebulin, several actins, myosins, and myomesins) could be detected in high abundance. Among the most abundant proteins we also found proteins directly related to muscle contraction (e.g., several tropomyosins and troponins), myoglobin (crucial for muscular oxygen supply), and several proteins involved in aerobic energy production and calcium handling (e.g., sarcoplasmic/endoplasmic reticulum calcium ATPase 2, calsequestrin, and the ryanodine receptor 2). Furthermore, we could detect highly relevant proteins of the DAPC (e.g., several sarcoglycans, dystroglycan, laminin alpha 2, dystrophin, dystrobrevin, several syntrophins and dystrophin), which are usually hard to detect by standard proteomics workflows (Murphy and Ohlendieck, 2016). To verify the reproducibility of the obtained data and to estimate the degree of proteome alterations, we used unsupervised hierarchical clustering (Figure 3A) and a principal-component analysis (Figure 3B) of the entire proteome profiles. With both methods, all four groups were well separated, which clearly demonstrates that age as well as *DMD* genotype significantly affect the heart muscle proteome. As already observed in skeletal muscle (Frohlich et al., 2016), changes related to heart muscle growth between 2 days and 3 months of age were by far more prominent than the differences between DMD and WT myocardium within both age groups.

### The Decreased Abundance of Several Mitochondrial Proteins Indicates an Impaired Mitochondrial Energy Production in the Myocardium of the 3-Month-Old DMD Pigs

The heart is known to be highly dependent on mitochondrial energy production by fatty acid β-oxidation and oxidative phosphorylation. Interestingly, several mitochondrial proteins were less abundant in the myocardium of the 3-month-old DMD compared with WT animals. Among them, 2,4-dienoyl-CoA reductase (DECR1), delta(3,5)-delta(2,4)-dienoyl-CoA isomerase (ECH1), and enoyl-CoA delta isomerase 1 (ECI1) are involved in β-oxidation, and NADH dehydrogenase [ubiquinone] flavoprotein 3 (NDUFV3) and NADH-ubiquinone oxidoreductase chain 1 (MT-ND1) are proteins of the respiratory chain. In addition, proteins from the mitochondrial membrane and matrix (e.g., glutaryl-CoA dehydrogenase [GCDH], cytochrome *b*5 type B [CYB5B], and sirtuin 3) were also less abundant, further indicating mitochondrial impairment in the myocardium of the DMD animals. However, compared with the previously observed dramatically reduced abundance of numerous proteins of glycolysis and oxidative phosphorylation in skeletal muscle of the same animals (Frohlich et al., 2016), negative effects on bioenergetic pathways seem to be less pronounced in the myocardium. Nevertheless, this finding is in line with previous proteomic findings of decreased abundance of mitochondrial proteins in the heart of *mdx* and *mdx-4cv* mice (Lewis et al., 2010; Murphy et al., 2016) and further supports the hypothesis that mitochondrial impairment is an important factor inducing DMD-related cardiomyopathy in humans (Meyers and Townsend, 2019).

### The Increased Abundance of Proteins Related to the Immune System May Reflect an Arising Inflammatory Pathology in the Myocardium of the 3-Month-Old DMD Pigs

Even though no gross histomorphological lesions and no specific alterations of fibrosis-associated proteins like fibronectin and collagens could be detected, the destabilization of heart cell membranes and resulting microtraumas may explain the increased abundance of several proteins related to inflammatory response in the heart samples of the 3-month-old DMD animals. As revealed by the DAVID analysis and visualized in Figure 5, proteins related to inflammation were predominantly more abundant in the 3-month-old DMD animals when compared with their 2-day-old counterparts. Among them were inter-alpha-trypsin inhibitor heavy chain H4 (ITIH4), alpha-1-acid glycoprotein 1 (ORM1), alpha-2-macroglobulin (A2M), and alpha-2-HS-glycoprotein (AHSG), all acute-phase proteins that are known to be increased in response to inflammation. Furthermore, proteins related to the immune system and wound healing like complement component C7 (C7), protein S100-A8 (S100A8), Toll-interacting protein (TOLLIP), glutathione peroxidase 1 (GPX1), and the platelet-activating factor acetylhydrolase (PLA2G7) were increased in the heart samples of 3-month-old DMD animals. However, the fact that no major proteome alterations related to cellular stress response and fibrosis were observed indicates that the myocardium of 3-month-old DMD animals reflects an arising but not the final stage of pathology caused by the cardiomyocyte's sarcolemma instability as a consequence of dystrophin loss.

### A Decreased Abundance of Ribosomal Proteins in Hearts of 2-Day-Old and 3-Month-Old DMD Animals May Reflect Reduced Translation Activity

The abundances of many ribosomal proteins were markedly decreased in myocardium from 2-day-old DMD pigs and, to a lesser extent, from 3-month-old DMD pigs when compared with the respective age-matched WT controls. Muscle protein synthesis rates are mainly determined by the cellular content of ribosomes, and an increase in ribosomal capacity and protein synthesis is required for muscle cell hypertrophy (Kim et al., 2019; Nader, 2014). The reduced abundance of ribosomal proteins in DMD myocardium thus argues for reduced translation activity and may explain the significantly reduced heart weight and cardiomyocyte diameters in 3-month-old DMD versus WT pigs. Nevertheless, the heart was less affected than the skeletal muscle as indicated by an increased relative heart weight (heart weight-to-body weight ratio) in 3-month-old DMD pigs (Figure 2A). In WT myocardium, a marked age-dependent decrease in the relative abundance of ribosomal proteins was observed in 3-month-old animals, most likely due to an increase in the amount of non-ribosomal, e.g., structural, proteins during growth of the cardiomyocytes and the entire heart. In DMD myocardium, the age-related decrease in the ribosomal protein content was less pronounced than in WT. This may be due to the facts that (1) the relative abundances of ribosomal proteins were already markedly decreased in 2-day-old animals and (2) cardiomyocyte hypertrophy and growth of the heart were reduced in DMD compared with WT pigs.

### The Decrease of Proteins from the Dystrophin-Associated Protein Complex May Indicate Further Destabilization of the Cardiomyocyte's Sarcolemma Ongoing with an Impaired Cellular Signaling

Beside dystrophin, SNTA1 was found to be decreased in the 2-day-old as well as in the 3-month-old DMD animals. SNTA1, expressed in striated muscle and heart, is known as a signal transducing adapter protein serving as a scaffold to a variety of signal proteins (for a review of syntrophins please refer to Bhat et al., 2019). In skeletal muscle, a decrease of SNTA1 was already demonstrated in biopsies of human patients with DMD (Wakayama et al., 2006). Furthermore, SNTA1 was shown to bind to spectrin-like repeats in dystrophin mediating the proper localization of nNOS (Adams et al., 2018). Concerning the heart, the PDZ domain of SNTA1 has been shown to bind cardiac voltage-gated sodium channels. Mutations in the *SNTA1* gene are known to be associated with a long QT syndrome (Hedley et al., 2009; Wu et al., 2008), which is like other long QT syndromes characterized by prolonged QT intervals and arrhythmias, which may result in sudden death. Besides dystrophin and SNTA1, further members of the DAPC were found to be less abundant in the 3-month-old DMD myocardium, among them being the three sarcoglycans (SGCA, SGCB and SGCD). SGCA, SGCB, and SGCD are members of the sarcoglycan transmembrane complex, which is known to be important for the linkage of extracellular components and is supposed to play an important role in the stability of muscle fibers (Allen et al., 2016; Constantin, 2014). Furthermore, dystrobrevin alpha (DTNA) was less abundant in the heart samples of the 3-month-old DMD animals. DTNA is supposed to play an important role in intracellular signaling, and DTNA deficiency in a mouse model led to moderate muscular dystrophy (Grady et al., 1999). Interestingly, *DTNA* mutations are associated with left ventricular non-compaction (Ichida et al., 2001). The IHC localization of dystrophin, SGCA, α-dystroglycan, and β-dystroglycan further confirmed that the formation of DAPC is severely impaired in the DMD animals. Taken together, the detected loss of important dystrophin complex components in the heart of the DMD animals indicates that both functions associated with this complex, signal transduction, and cell membrane stabilization may be impaired and may cause a severe myopathy.

### The Altered Abundance of Several Proteins Directly Related to Heart Muscle Function May Mirror an Early-Stage Cardiomyopathy

Notably, several proteins playing an important role in heart muscle function are altered in abundance in the heart of DMD pigs. For instance, compared with the age-matched controls, myosin-6 (MYH6) was strongly reduced in the 2-day-old and 3-month-old animals (10-fold in 2-day- and 15-fold in 3-month-old animals). MYH6, frequently referred to as myosin heavy chain α (MHC-alpha), is one of the major proteins of the cardiac muscle thick filament and is directly involved in muscle contraction. Mutations in the *MYH6* gene are associated with hypertrophic as well as dilated cardiomyopathy (Carniel et al., 2005; Niimura et al., 2002), frequently associated with DMD (Kamdar and Garry, 2016). A further protein, related to MHC-alpha, the myosin regulatory light chain 2 (MYL7), frequently referred to as atrial light chain-2 (ALC-2), was also found to be reduced in the myocardium of 2-day-old DMD animals. Although the complete functional pattern of this protein remains unclear, it is supposed that MYL7 is a player in cardiac development and contractility. In baboons, levels of ALC-2 expression were shown to be correlated to the expression levels of MHC-alpha in the atrial myocardium (Henkel et al., 1993). Strikingly, the abundance alteration of MYL7 switched during the development of the DMD pigs. MYL7 was found to be less abundant in the heart of 2-day-old DMD animals (8.9-fold), whereas it was more abundant in the 3-month-old DMD animals (1.6-fold), which may hint to a compensatory function of this protein in the older DMD animals. This was confirmed by IHC, where MYL7 could be localized in Purkinje fibers and most cardiomyocytes, but was more intense in the cardiomyocytes of the 3-month-old DMD versus WT animals. Furthermore, an increase (4.9 fold) of MYL4, often referred to as atrial light chain-1 (ALC-1), was observed in the myocardium of 3-month-old DMD animals. Interestingly, IHC revealed that in WT animals MYL4 expression was restricted to Purkinje fibers, whereas some cardiomyocytes of DMD animals exhibited MYL4 expression, predominantly near the endocardial site and occasionally scattered within the ventricle. MYL4 is supposed to be important for heart muscle function and to increase contractility (Morano et al., 1996). In humans, it was demonstrated that MYL4 expression decreases in cardiomyocytes during the first years of life, which leads to a mosaic expression (Wang et al., 2018). This decrease in abundance seems also to be the case in the porcine system where we found a massive decrease (24-fold) of MYL4 in 3-month-old compared with 2-day-old WT animals. Interestingly, in patients with dilated and hypertrophic cardiomyopathy, a significant increase in MYL4 was observed (Morano et al., 1997; Ritter et al., 1999), and it was hypothesized that this upregulation may be part of a compensatory mechanism. In addition, an increase of keratin 8 (KRT8) and keratin 19 (KRT19) was observed in the heart of DMD animals of both age groups. KRT8 and KRT19 are supposed to be involved in the linkage of the contractile apparatus to dystrophin (Stone et al., 2005), which may also be a compensatory effect.

### The Degree of Proteome Alterations and the Affected Biochemical Pathways in Heart and Skeletal Muscle Are Very Different

**Figure 8:**
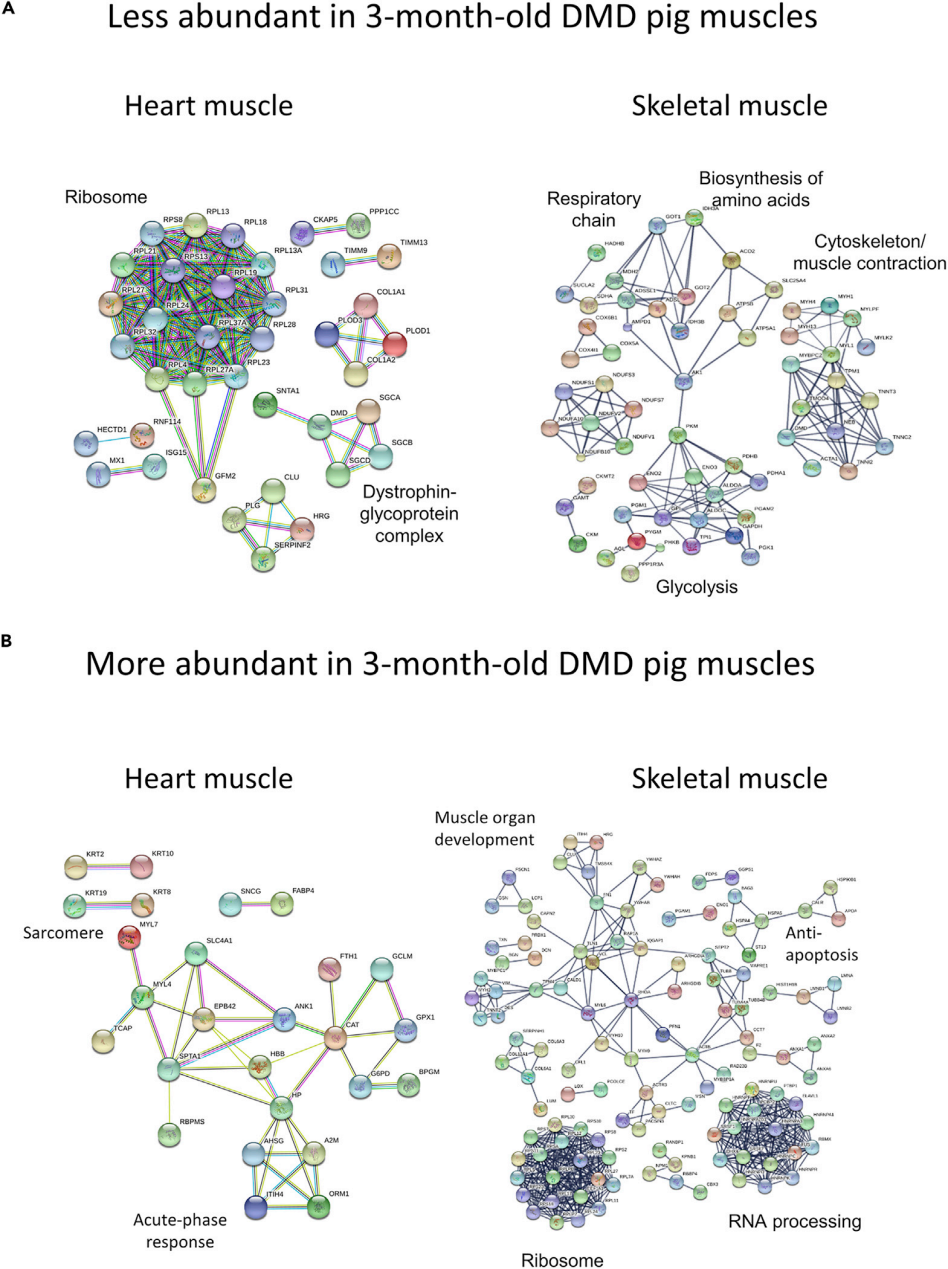
Comparative STRING Analysis of Differentially Abundant Proteins from Heart and Skeletal Muscle (A and B) (A) Proteins that are less abundant and (B) proteins that are more abundant in DMD muscles. For the analysis of skeletal muscle, the data from Frohlich et al. (2016) were used.

By comparing the DMD with WT proteome profiles, we were able to detect prominent differences in both age groups. However, we found that compared with the 2-day-old animals, the number of differentially abundant proteins in the 3-month-old DMD animals was not increased (see Figure 4B). This is surprising because in skeletal muscle of the same animals, the increase of proteome derangements from 2-day- to 3-month-old animals was massive (Frohlich et al., 2016). A direct comparison of the degree of proteome alteration for skeletal muscle and heart datasets is given in Table 1. Histopathological examination of the porcine DMD model revealed severe dystrophic lesions in skeletal muscle (Klymiuk et al., 2013), but not in the left ventricular myocardium of the 3-month-old animals. This reflects the situation in human patients with DMD where cardiomyopathies usually occur at later stages. Interestingly, not only the degree of proteomic alterations in DMD versus WT but also the biochemical pathways that were affected were different between skeletal and heart muscle. To illustrate this, STRING analysis of the differentially abundant proteins of heart and skeletal muscle (Frohlich et al., 2016) from the 3-month-old animals is shown in Figure 8. For example, in skeletal muscle of DMD pigs, a broad fraction of proteins related to energy production and myosins was found to be less abundant, whereas in the myocardium, a lot of ribosomal proteins were found to be less abundant than in WT animals (Figure 8A). Conversely, a broad fraction of these ribosomal proteins was found to be more abundant in the skeletal muscle, whereas several proteins related to immunological processes were more abundant in heart muscle (Figure 8B). This finding clearly indicates that DMD progression in the myocardium is not only slower than in skeletal muscle but also involves different biological and biochemical pathways.

**Table 1:** Degree of Proteome Alterations Detected in Skeletal Muscle (Frohlich et al., 2016) and Heart Muscle

Quantified Proteins	Heart Muscles			Skeletal Muscles		
	2,450			1,428		
	Diff. Abundant	More Abundant	Less Abundant	Diff. Abundant	More Abundant	Less Abundant
2-day-old DMD versus WT	190 **(7.8%)**	37 **(1.5%)**	153 **(6.2%)**	53 **(3.7%)**	15 **(1.0%)**	38 **(2.7%)**
3-month-old DMD versus WT	147 **(6.0%)**	55 **(2.2%)**	92 **(3.8%)**	337 **(23.6%)**	235 **(16.5%)**	102 **(7.1%)**

To facilitate the comparison between both datasets, the degree of abundance alterations is expressed in percent of quantified proteins.

### Concluding Remarks

The proteome study of myocardium from a clinically severe pig model for DMD detected a variety of stage-specific proteome aberrations induced by the absence of dystrophin. Many of the findings fit to what is known about cardiomyopathies during DMD progression and provide new insights into the underlying biochemistry. Furthermore, when compared with data from skeletal muscle from the same individuals, our data clearly demonstrate that DMD pathology in heart and skeletal muscle is not only different concerning onset and velocity but also according to the involved biochemical pathways, which further underlines the complexity of the disease. The detected proteome alterations and biochemical pathways are particularly valuable, as they can serve as readouts for the efficacy of new therapeutic approaches in preclinical models.

### Limitations of the Study

In this study, we analyzed disturbances in the myocardial proteome of a pig model for DMD. Although the porcine DMD model reflects the human pathology, there may be intrinsic differences, e.g., related to the rapid growth of pigs compared with humans. Therefore, conclusions of the presented results should be drawn with caution and should be used as a starting point for further investigations in human DMD samples. Moreover, it has to be considered that even though MS-based proteomics has become a powerful tool for biomedical research, its analytical depth is still limited and that several biochemical pathways playing a role in DMD may have not been detected. Finally, it has to be considered that changes in protein abundance alone cannot characterize the entire pathology of a complex disease like DMD.

### Resource Availability

#### Lead Contact

Requests should be addressed to the Lead Contact, Dr. Thomas Fröhlich, frohlich@genzentrum.lmu.de.

#### Materials Availability

New materials were not generated in this study.

#### Data and Code Availability

The mass spectrometry proteomics data have been deposited to the ProteomeXchange Consortium (Vizcaino et al., 2014) via the PRIDE partner repository with the dataset identifier PXD019413.

## Methods

All methods can be found in the accompanying Transparent Methods supplemental file.

## Supplemental Information

Document S1. Transparent Methods and Figures S1–S6

Table S1. List of Identified Proteins; Related to Figure 3

Table S2. Lists of Proteins with Age-Dependent Abundance Changes in WT Animals; Related to Figure 4

Table S3. Lists of Proteins with Age-Dependent Abundance Changes in DMD Animals; Related to Figure 4

Table S4. Lists of Proteins with Abundance Changes in 2-Day-Old DMD versus WT Animals; Related to Figure 4

Table S5. Lists of Proteins with Abundance Changes in 3-Month-Old DMD versus WT Animals; Related to Figure 4

Table S6. DAVID Analysis of Proteins Less Abundant in 2-Day-Old DMD versus WT Animals; Related to Figure 5

Table S7. DAVID Analysis of Proteins More Abundant in 2-Day-Old DMD versus WT Animals; Related to Figure 5

Table S8. DAVID Analysis of Proteins Less Abundant in 3-Month-Old DMD versus WT Animals; Related to Figure 5

Table S9. DAVID Analysis of Proteins More Abundant in 3-Month-Old DMD versus WT Animals; Related to Figure 5
