# Supplementary material for: Progressive Proteome Changes in the Myocardium of a Pig Model for Duchenne Muscular Dystrophy
Source: iScience. 2020 Sep 1;23(9):101516. doi: 10.1016/j.isci.2020.101516 (PMC7495112; doi:10.1016/j.isci.2020.101516)
Supplement: Document S1. Transparent Methods and Figures S1–S6 [file mmc1.pdf]

iScience, Volume *23*

Supplemental Information

Progressive Proteome Changes in the Myocardium of a Pig Model for Duchenne Muscular Dystrophy

Hathaichanok Tamiyakul, Elisabeth Kemter, Miwako Kösters, Stefanie Ebner, Andreas Blutke, Nikolai Klymiuk, Florian Flenkenthaler, Eckhard Wolf, Georg J. Arnold, and Thomas Fröhlich

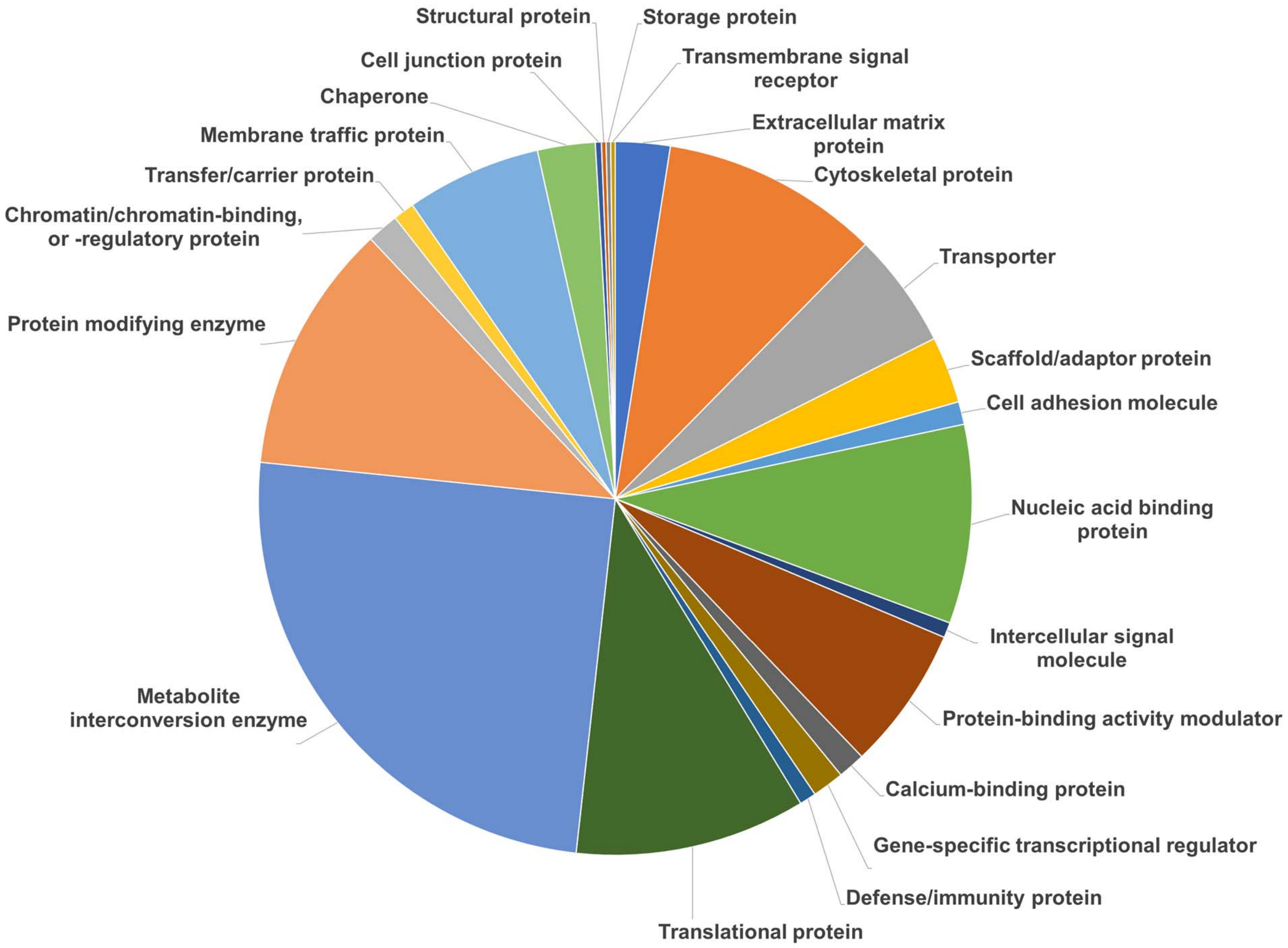

Supplementary Figure 1 (related to Figure 3): Pie chart summarizing PANTHER protein classes of myocardial proteins identified by mass spectrometry

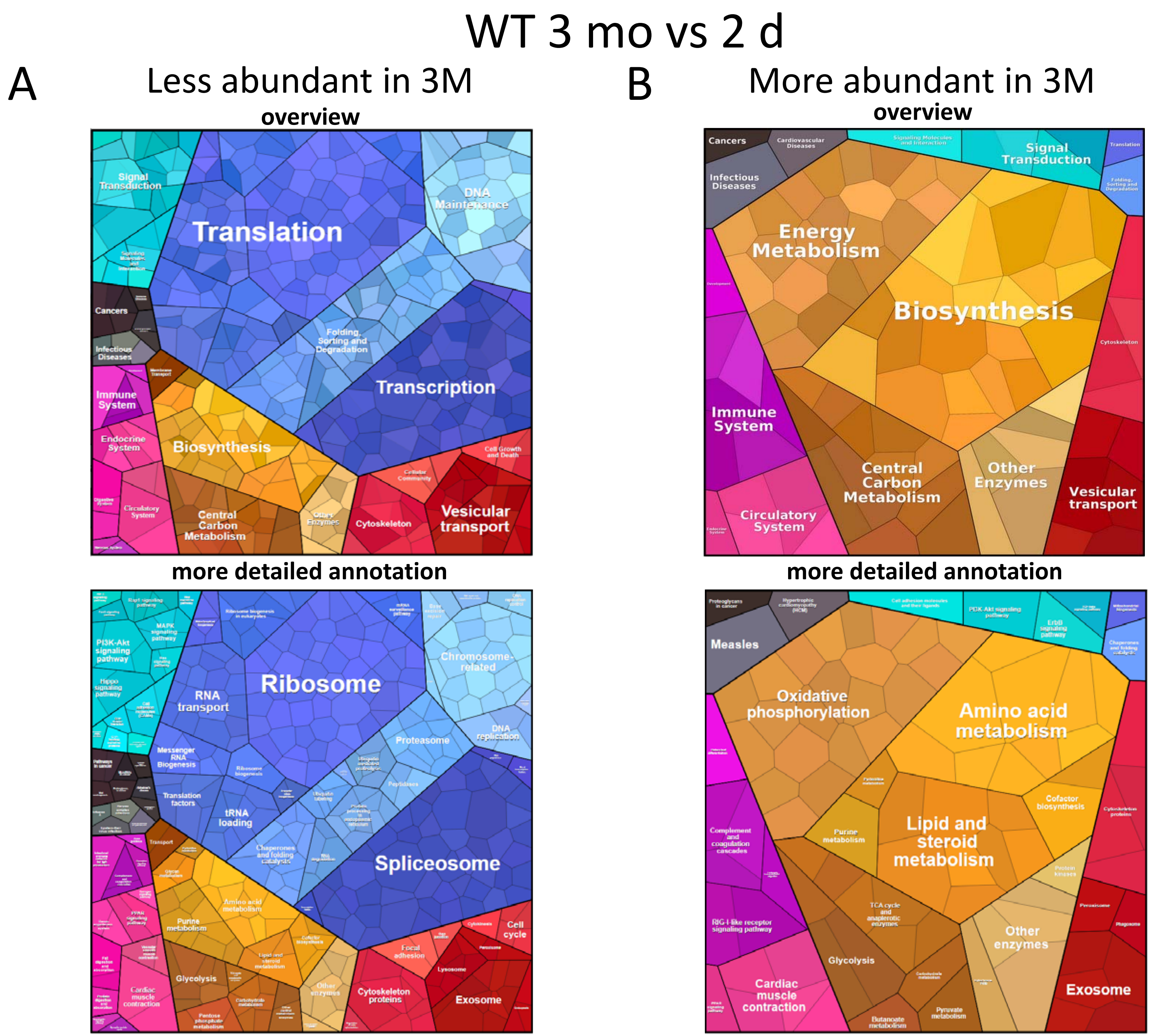

Supplementary Figure 2 (related to Figure 4): Proteomaps analysis of proteins which were differently abundant between 3-month-old and 2-day-old WT myocardium.

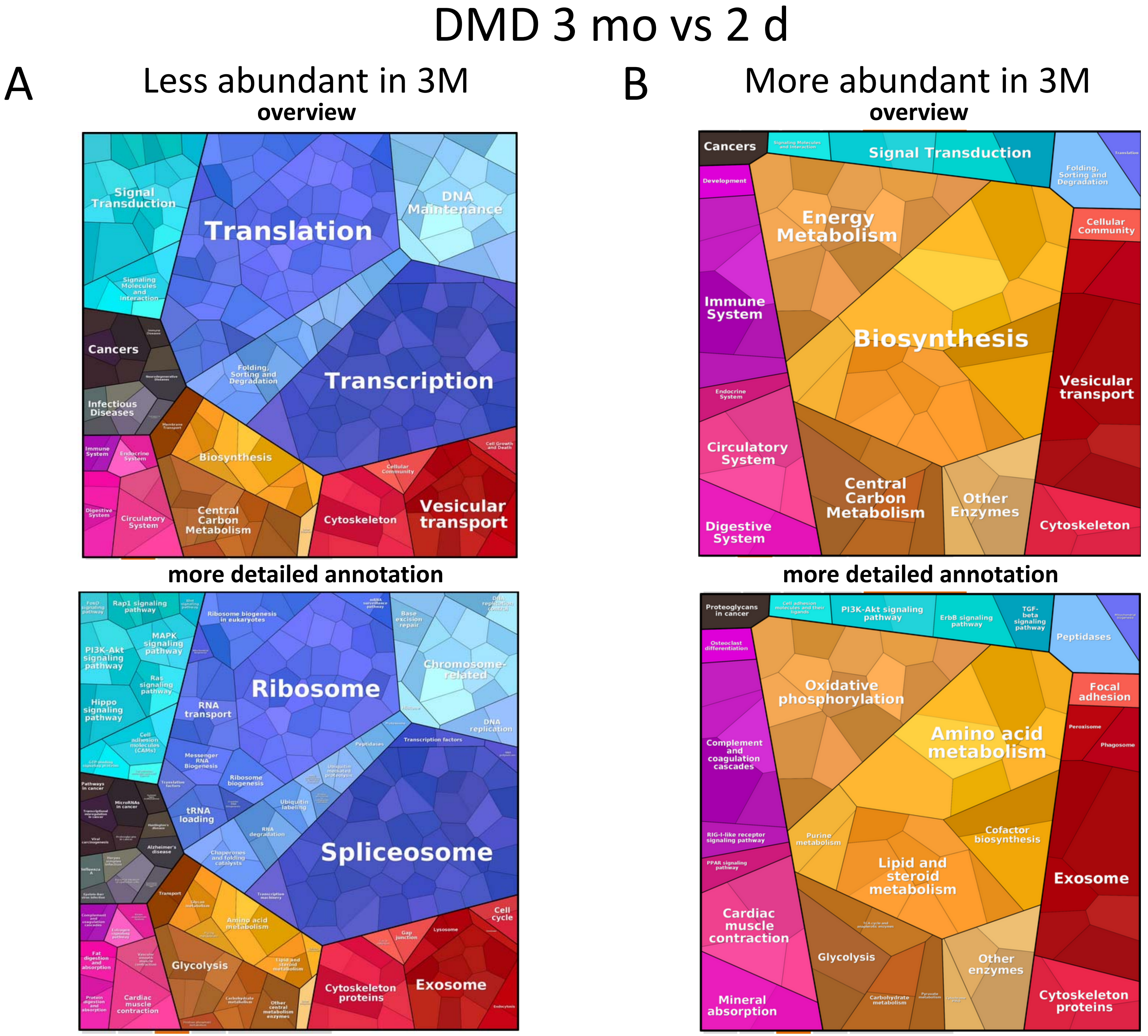

Supplementary Figure 3 (related to Figure 4): Proteomaps analysis of proteins which were differently abundant between 3-month-old and 2-day-old DMD myocardium.

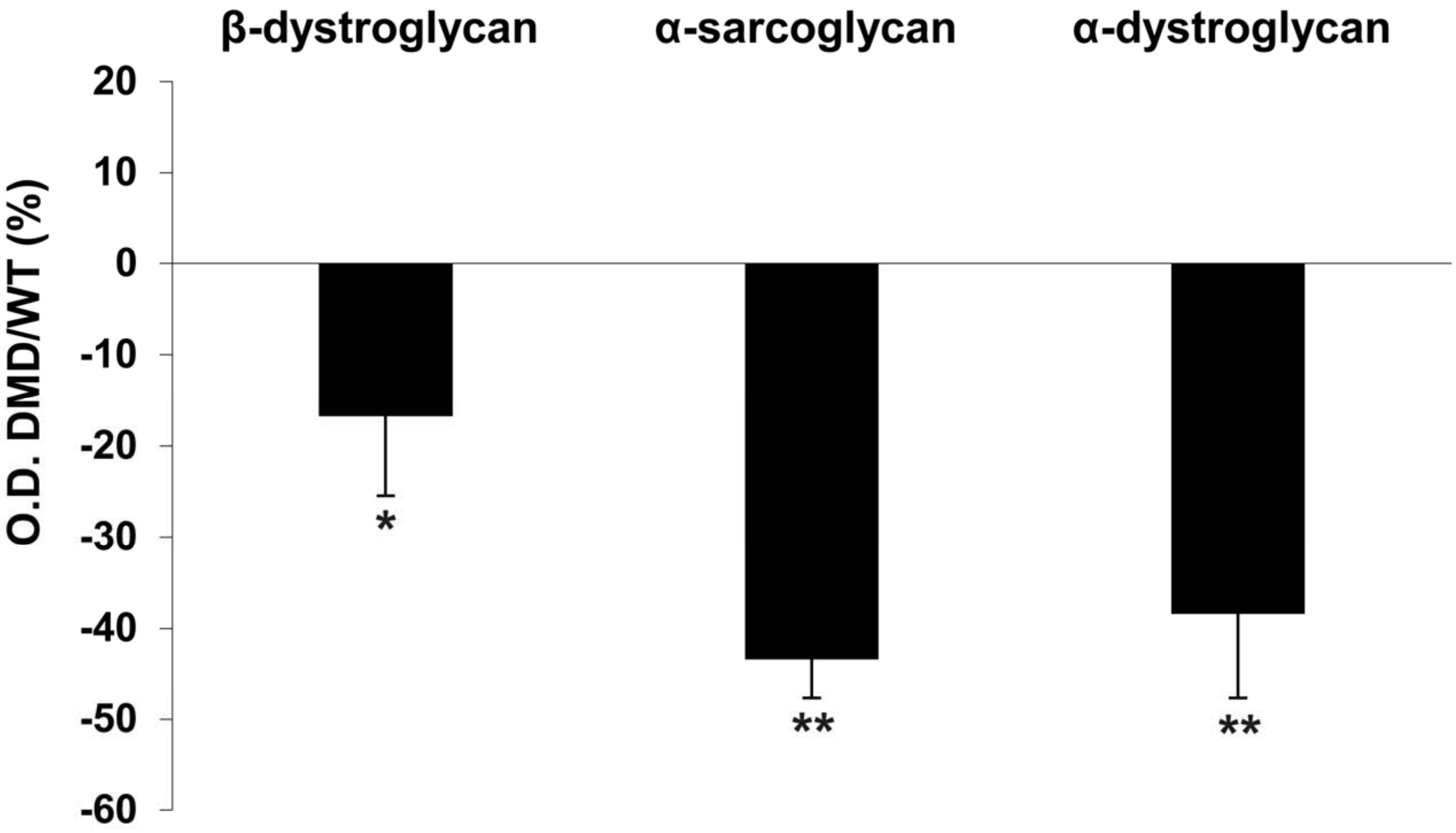

Supplementary Figure 4 (related to Figure 6): IHC-signal intensity of members of the dystrophin-associated protein complex (DAPC) in the myocardium of 3-month-old DMD and WT pigs. IHC-signal intensities were measured as the optical density (OD) of myocardial IHC-sections of WT and DMD pigs and are shown as the percentual difference of the IHC-image ODs in DMD *vs.* WT sections. Significant OD differences between DMD and WT sections (n=3) are indicated by asterisks: *: $p< 0.05$; **: $p< 0.01$ (Student's t-test).

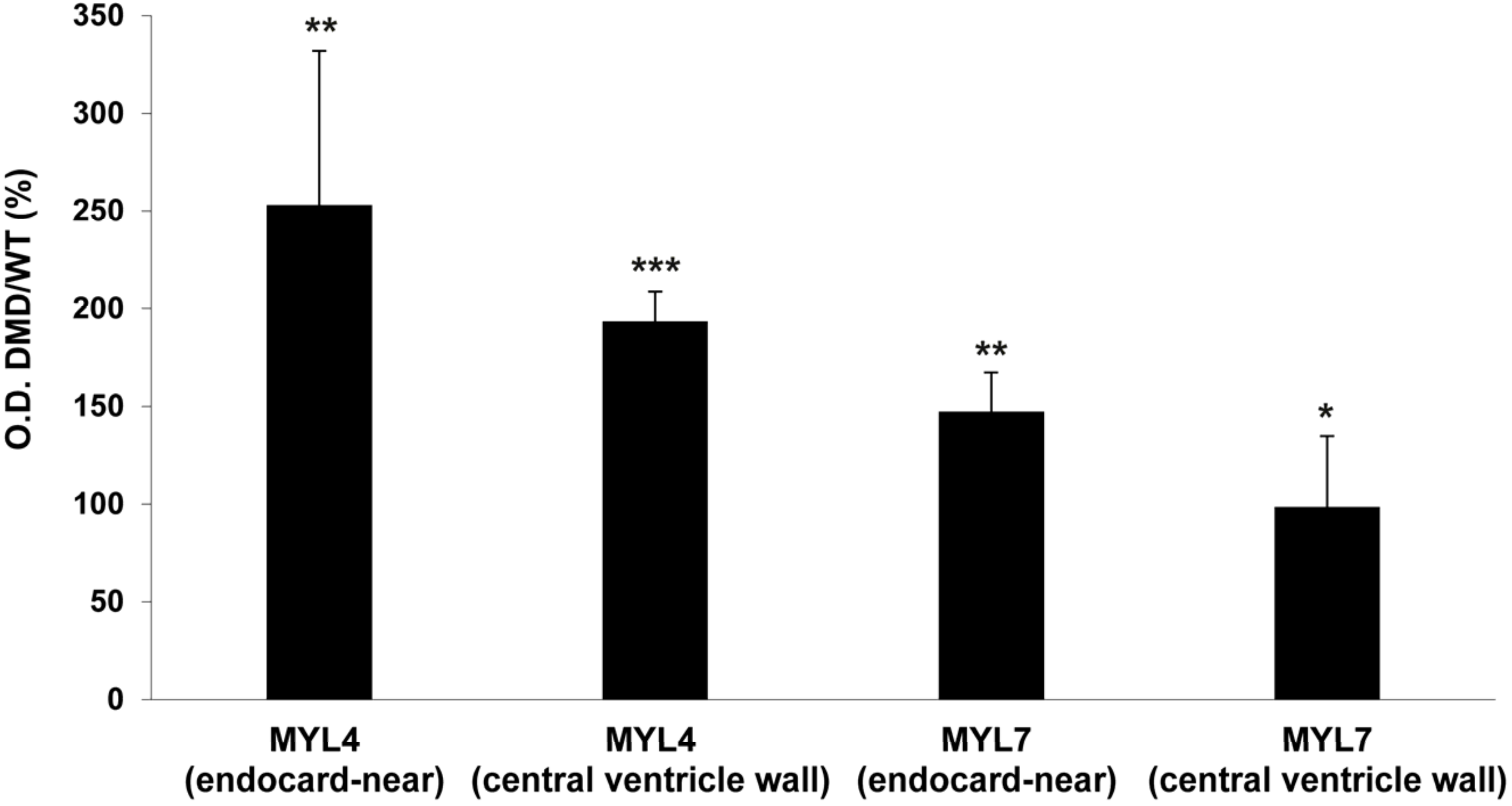

Supplementary Figure 5 (related to Figure 7): IHC-signal intensity of MYL4 and MYL7 in the heart of 3-month-old DMD and WT pigs. IHC-signal intensities were measured as the optical density (OD) of myocardial IHC-sections of WT and DMD pigs and are shown as the percentual difference of the IHC-image ODs in DMD *vs.* WT sections. Significant OD differences between DMD and WT sections (n=3) are indicated by asterisks: *: $p < 0.05$; **: $p < 0.01$; ***: $p < 0.01$ (Student's t-test).

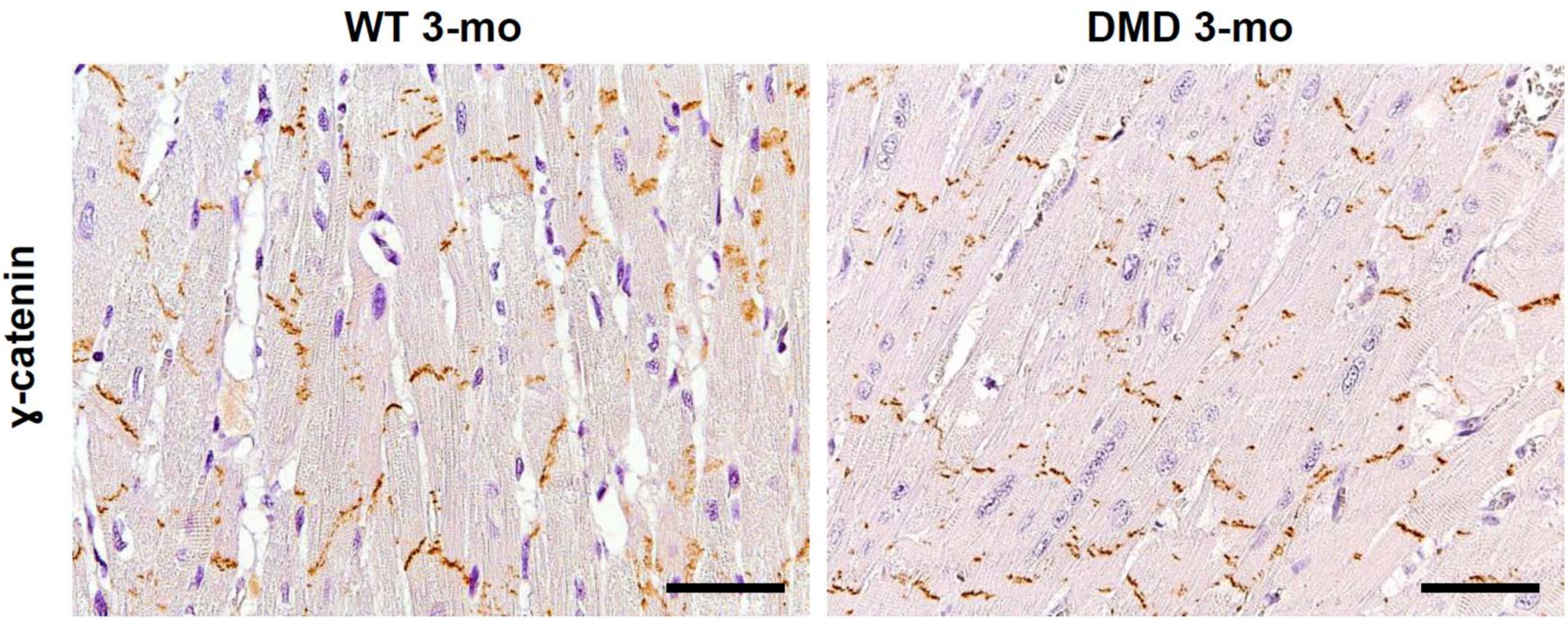

Supplementary Figure 6 (related to Figure 7): Immunohistochemical detection of γ-catenin (brown color) to demonstrate intercalated discs of cardiomyocytes in myocardium of 3-month-old DMD and WT pigs (Paraffin sections). 3,3'-Diaminobenzidine (DAB) was used as chromogen, and hemalum as nuclear counterstain. Bars = 50 µm.

Transparent Methods

General chemical reagents

Analytical grade urea, dithioerythritol (DTE), trichloroacetic acid and Tris was purchased from Carl Roth GmbH + Co. KG (Karlsruhe, Germany). Formic acid, acetonitrile and water was obtained from Merck KGaA (Darmstadt, Germany). CHAPS was purchased from Sigma-Aldrich (St. Louis, MI, USA)

Animals and tissue samples

DMD pigs were generated as described before (Klymiuk et al., 2013). All animal experiments were carried out in accordance with the German Animal Welfare Act and were approved by the responsible animal welfare authority (District Government of Upper Bavaria, Reference Number 55.2-1-54-2531-86-10). Each three male DMD piglets and three age- and sex-matched wild-type (WT) control animals were euthanized at 2 days of age and at 3 months of age. The body weights of all examined pigs were recorded. In 3-month-old DMD and WT pigs, the absolute and relative heart weights were determined as well. For proteomic analyses, myocardial tissue specimen of approximately 200 mg were sampled from the parietal papillary muscle of the left ventricle, shock frozen on dry ice, and stored at −80 °C until further analysis. Additional samples were fixed in neutrally buffered formaldehyde solution (4%) for 24 hours, routinely processed and embedded in paraffin or in plastic [glycol methacrylate and methyl methacrylate (GMA/MMA)], as described previously (Frohlich et al., 2016; Klymiuk et al., 2013).

Histopathology and quantitative histomorphological analyses

Histopathological examinations were performed on standard hematoxylin and eosin (HE) stained paraffin sections. The mean minimal muscle fiber (Feret) diameters (Klymiuk et al., 2013) were determined in left-ventricular papillary muscle samples of 3-month-old DMD and WT pigs. To avoid bias introduced by the anisotropic orientation of muscle fibers, muscle fiber diameters were determined in GMA/MMA-embedded “orthogonal triplet sections” (ORTRIPS) (Albl et al., 2016; Mattfeldt et al., 1985), generated as described earlier (Albl et al., 2016). First, an isotropic uniform random (IUR) sample section plane was generated, using the so-called “orientator” method (Albl et al., 2016; Mattfeldt et al., 1990). Then a second and a third section plane were cut perpendicular to

the IUR section plane and to each other. The ORTRIP sections were embedded in GMA/MMA, sectioned and stained with HE. The mean minimal muscle fiber diameters were determined in systematically randomly sampled fields of view (33 ±9 per case) at 630 x magnification, superimposed with unbiased counting frames (Howard and Reed, 2005), using a Videoplan^{TM} image analysis system (Zeiss-Kontron, Augsburg, Germany). Per case, 354 ±67 muscle fiber section profiles were measured.

Sample preparation for proteomic analysis

To 2 mg of tissue sample 100 µL of lysis buffer consisting of 8 M urea, 4% CHAPS, 40 mM Tris and 65 mM DTE was added and lysed using a homogenizer (Art-Miccra D-8, Miccra GmbH, Müllheim, Germany) and QIAshredder devices (Qiagen, Hilden, Germany). Protein concentrations were determined using the Pierce 660 nm protein assay (Thermo Fisher Scientific, Waltham, MA, USA). Prior to digestion, a trichloroacetic acid precipitation was performed to remove interfering CHAPS.

Tryptic digestion, iTRAQ-labeling and OFFGEL pre-fractionation

Proteins were dissolved, reduced and cysteines were blocked as described in the iTRAQ reagent kit's protocol (SCIEX, Framingham, MA, USA). Digestion was performed using porcine trypsin at an enzyme to protein ratio of 1/50 (Promega, Madison, WI, USA) and incubation overnight at 37 °C. Tryptic peptides from WT-2D, DMD-2D, WT-3M and DMD-3M were labeled with 114, 115, 116 and 117 reagents, respectively and pooled. As suggested by the iTRAQ kit manufacturer, pooled samples were purified using cation-exchange chromatography (cartridge system, SCIEX) and desalted using Pierce C18 spin columns (Thermo Fisher Scientific). For preparative isoelectric focusing, the OFFGEL system (3100 OFFGEL Fractionator, Low Res Kit, pH 3–10, Agilent, Santa Clara, CA, USA) was used as described in the instruments manual. Prior to LC–MS/MS analysis, samples were again desalted using C18 spin columns (Pierce, Thermo Scientific, Rockford, IL, USA).

Liquid chromatography mass spectrometry (LC-MS)

For LC-MS analysis, an Ultimate 3000 nano-LC system (Thermo Fisher Scientific) coupled to a Triple TOF 5600 mass spectrometer (SCIEX) was used. Peptides (2-10 µg) were diluted in 15 µL 0.1% formic acid (FA) and injected on a trap column (30 µm x 10 cm

nanoViper, Thermo Fisher Scientific) at a flowrate of 30 µL/min. Separation was done using a 50 cm column (Acclaim PepMap RSLC, 75 µm x 50 cm nanoViper, C18, 2 µm, 100A, Thermo Fisher Scientific) at a flowrate of 200 nL/min using 0.1% FA as solvent A and 0.1% FA in acetonitrile as solvent B. The method consisted of the following multi-step gradient: 5% to 25 % B in 290 min and 25% B to 50 % B in 30 min followed by a 15 min elution step at 84% B. MS acquisition was performed in the data dependent mode using a top 70 method and the following conditions: IonSpray Voltage: 2.3 kV, MS Scan: 400 – 1250 m/z, CID MS/MS scans: 100-1800 m/z.

Data processing and bioinformatics

From SCIEX wiff files, Mascot generic files were generated using the AB SCIEX MS converter V4.1 and searched using MASCOT V2.6.1 and the Sus scrofa subset of the NCBI refseq database. For identification, the following parameters were used: i) Enzyme: Trypsin; ii) Fixed modifications: Methylthio (C); iii) Variable modifications: Oxidation (M); iv) Peptide charge: 2+, 3+ and 4+; v) Peptide tol. ±: 50 ppm; vi) MS/MS tol. ±: 0.1 Da; vii) Quantitation: iTRAQ 4plex. Identified proteins were classified according to PANTHER protein classes using PantherDB V15.0 (http://www.pantherdb.org) (Mi et al., 2019). For iTRAQ signal quantification and statistical evaluation, Scaffold V4.8.9 (Proteome Software, Portland, OR, USA) was used. Protein identifications with at least 2 individual peptides were filtered for an FDR < 1%. For iTRAQ-based quantification, Scaffold standard parameters were used and, as recommended for iTRAQ experiments, statistical evaluation was performed using Permutation Test and Benjamini-Hochberg multiple test correction. Differences with a minimum log2 fold-change of +/- 0.6 were regarded as relevant. For hierarchical clustering and principle component analysis, Log2 normalized iTRAQ intensity values and Perseus V1.5.3.2 (Tyanova et al., 2016) were used. Proteins affected in abundance were functionally characterized and clustered using Proteomaps (Liebermeister et al., 2014), DAVID (Huang da et al., 2009) and STRING (Szklarczyk et al., 2019).

Immunohistochemistry and digital image analysis

Fixation of heart samples in neutrally buffered formaldehyde solution (4%), paraffin embedding and sectioning and histological analyses of heart samples were performed as

described previously (Kemter et al., 2017). Immunohistochemistry on formalin-fixed tissue slices after heat-induced antigen retrieval in citrate buffer (pH 6) and blocking of endogenous peroxide by 1% H_2O_2 treatment was performed using the following primary antibodies: mouse monoclonal antibody against γ-catenin (1:50, clone A-6, no. sc-514115, Santa Cruz Biotechnology, Santa Cruz, CA, USA), mouse monoclonal antibody against DYS1 (1:100, clone DY4/6D3, no. NCL-DYS1, Leica biosystems, Wetzlar, Germany), mouse monoclonal antibody against α-dystroglycan (1:60, clone IIH6, no. sc-53987, Santa Cruz Biotechnology), mouse monoclonal antibody against β-dystroglycan (1:150, clone 7D11, no. sc-33701, Santa Cruz Biotechnology), and rabbit polyclonal antibody against MYL7 (1:200, no. HPA013331, Sigma-Aldrich). Heat-induced antigen retrieval in Tris-EDTA buffer (pH 9) was applied for immunohistochemistry using following primary antibodies: mouse monoclonal antibody against MYL4 (1:2500, clone OTI2F6, no. TA807581, Origene, Rockville, MD, US), and mouse monoclonal antibody against α-sarcoglycan (1:100, clone AD1/20A6, no. NCL-L-a-SARC, Leica biosystems). As secondary antibody, a biotinylated polyclonal goat anti-rabbit IgG antibody (1:200, no. BA-1000, Vector Laboratories, Burlingame, CA, USA) was used for MYL7, a biotinylated polyclonal goat anti-mouse IgM antibody (1:100, no. BA-2020, Vector Laboratories) was used for α-dystroglycan, and a biotinylated polyclonal goat anti-mouse IgG antibody (1:500, no. 115-065-146, Jackson Immuno Research, West Grove, PA, USA) for all other immunohistochemistry reactions. After incubation with horse radish peroxidase (HRP)-linked avidin-biotin complexes (no. PK-6100, Vector Laboratories), immunoreactivity was visualized using 3,3-diaminobenzidine tetrahydrochloride dihydrate (DAB) (brown color). Nuclear counterstaining was done with hemalum (blue color). IHC-signal intensities were quantified by digital image analysis, using ImageJ (Schneider et al., 2012). IHC-staining intensities of α-sarcoglycan, α-dystroglycan, β-dystroglycan, MYL4 and MYL 7 were analyzed by optical density (OD) measurements in digital images acquired at 10 x objective magnification, taken in nine randomly sampled section locations per case. Digital images were processed for standardized white-reference background correction (entire image adjustment), using the Microsoft Office 2010 Picture Manager. OD-measurements were performed according to the ImageJ User Guide (Edition IJ 1.46r; https://documents.pub/document/imagej-manual.html). Image analysis data are

presented as the mean percentual OD differences of IHC-sections from DMD *vs.* WT samples.

Supplemental References

Albl, B., Haesner, S., Braun-Reichhart, C., Streckel, E., Renner, S., Seeliger, F., Wolf, E., Wanke, R., and Blutke, A. (2016). Tissue Sampling Guides for Porcine Biomedical Models. Toxicol Pathol *44*, 414-420.

Frohlich, T., Kemter, E., Flenkenthaler, F., Klymiuk, N., Otte, K.A., Blutke, A., Krause, S., Walter, M.C., Wanke, R., Wolf, E.*, et al.* (2016). Progressive muscle proteome changes in a clinically relevant pig model of Duchenne muscular dystrophy. Sci Rep *6*, 33362.

Howard, C.V., and Reed, M.G. (2005). Unbiased Stereology, 2 edn (Coleraine, UK: QTP Publications).

Huang da, W., Sherman, B.T., and Lempicki, R.A. (2009). Systematic and integrative analysis of large gene lists using DAVID bioinformatics resources. Nat Protoc *4*, 44-57.

Kemter, E., Frohlich, T., Arnold, G.J., Wolf, E., and Wanke, R. (2017). Mitochondrial Dysregulation Secondary to Endoplasmic Reticulum Stress in Autosomal Dominant Tubulointerstitial Kidney Disease - UMOD (ADTKD-UMOD). Sci Rep *7*, 42970.

Klymiuk, N., Blutke, A., Graf, A., Krause, S., Burkhardt, K., Wuensch, A., Krebs, S., Kessler, B., Zakhartchenko, V., Kurome, M.*, et al.* (2013). Dystrophin-deficient pigs provide new insights into the hierarchy of physiological derangements of dystrophic muscle. Hum Mol Genet *22*, 4368-4382.

Liebermeister, W., Noor, E., Flamholz, A., Davidi, D., Bernhardt, J., and Milo, R. (2014). Visual account of protein investment in cellular functions. Proc Natl Acad Sci U S A *111*, 8488-8493.

Mattfeldt, T., Mall, G., Gharehbaghi, H., and Moller, P. (1990). Estimation of surface area and length with the orientator. J Microsc *159*, 301-317.

Mattfeldt, T., Mobius, H.J., and Mall, G. (1985). Orthogonal triplet probes: an efficient method for unbiased estimation of length and surface of objects with unknown orientation in space. J Microsc *139*, 279-289.

Mi, H., Muruganujan, A., Ebert, D., Huang, X., and Thomas, P.D. (2019). PANTHER version 14: more genomes, a new PANTHER GO-slim and improvements in enrichment analysis tools. Nucleic Acids Res *47*, D419-D426.

Schneider, C.A., Rasband, W.S., and Eliceiri, K.W. (2012). NIH Image to ImageJ: 25 years of image analysis. Nat Methods *9*, 671-675.

Szklarczyk, D., Gable, A.L., Lyon, D., Junge, A., Wyder, S., Huerta-Cepas, J., Simonovic, M., Doncheva, N.T., Morris, J.H., Bork, P.*, et al.* (2019). STRING v11: protein-protein association networks with increased coverage, supporting functional discovery in genome-wide experimental datasets. Nucleic Acids Res *47*, D607-D613.

Tyanova, S., Temu, T., Sinitcyn, P., Carlson, A., Hein, M.Y., Geiger, T., Mann, M., and Cox, J. (2016). The Perseus computational platform for comprehensive analysis of (prote)omics data. Nat Methods *13*, 731-740.
